# Gadd45g initiates embryonic stem cell differentiation and inhibits breast cell carcinogenesis

**DOI:** 10.1038/s41420-021-00667-x

**Published:** 2021-10-02

**Authors:** Xinbao Zhang, Yuting Li, Junxiang Ji, Xin Wang, Meng Zhang, Xiangfen Li, Yan Zhang, Zhenhua Zhu, Shou-Dong Ye, Xiaoxiao Wang

**Affiliations:** 1 Center for Stem Cell and Translational Medicine, School of Life Sciences, Anhui University, 230601 Hefei, Anhui P.R. China; 2 Institute of Physical Science and Information Technology, Anhui University, 230601 Hefei, Anhui P.R. China; 3 The First Affiliated Hospital of USTC, Division of Life Sciences and Medicine, University of Science and Technology of China, 230001 Hefei, Anhui P.R. China

**Keywords:** Embryonic stem cells, Cell signalling

## Abstract

Many self-renewal-promoting factors of embryonic stem cells (ESCs) have been implicated in carcinogenesis, while little known about the genes that direct ESCs exit from pluripotency and regulate tumor development. Here, we show that the transcripts of Gadd45 family genes, including Gadd45a, Gadd45b, and Gadd45g, are gradually increased upon mouse ESC differentiation. Upregulation of Gadd45 members decreases cell proliferation and induces endodermal and trophectodermal lineages. In contrast, knockdown of Gadd45 genes can delay mouse ESC differentiation. Mechanistic studies reveal that Gadd45g activates MAPK signaling by increasing expression levels of the positive modulators of this pathway, such as Csf1r, Igf2, and Fgfr3. Therefore, inhibition of MAPK signaling with a MEK specific inhibitor is capable of eliminating the differentiation phenotype caused by Gadd45g upregulation. Meanwhile, GADD45G functions as a suppressor in human breast cancers. Enforced expression of GADD45G significantly inhibits tumor formation and breast cancer metastasis in mice through limitation of the propagation and invasion of breast cancer cells. These results not only expand our understanding of the regulatory network of ESCs, but also help people better treatment of cancers by manipulating the prodifferentiation candidates.

## Introduction

Embryonic stem cells (ESCs) isolated from the inner cell mass of preimplantation blastocysts have two important characteristics, self-renewal and pluripotency [1]. In 1981, Evans and Martin et al. established ESCs in mice for the first time [2, 3]. The undifferentiated state of mouse ESCs (mESCs) can be maintained under serum-containing medium supplemented with LIF or in serum-free medium by adding 2i, including MEK inhibitor PD0325091 (PD03) and Gsk3β inhibitor CHIR99021 (CHIR) [4–6]. 2i/LIF-mediated FGF/MEK/ERK, Wnt/β-catenin, and LIF/STAT3 signaling pathways have become important pathways to regulate naïve pluripotency. To date, self-renewal circuitry in mESCs is increasingly defined. Oct3/4, Sox2, and Nanog are the core transcription factors that safeguard the stemness of ESCs [1]. Additionally, many genes with the ability to promote self-renewal have also been identified, such as Tfcp2l1, Klf2, Klf4, and Esrrb [7–11]. Interestingly, many self-renewal genes have a robust function in promoting cancer cell carcinogenesis. For instance, Oct4, Sox2, or Nanog upregulation facilitates the expansion features of breast cancer stem cells and promotes breast cancer tumorigenesis [12–14], and KLF family members have always been recognized as oncogenes in multiple tumors [15]. However, little is known about the mechanism events that drive the differentiation processes of ESCs, and what is the specific function of them during tumor cell carcinogenesis.

Growth arrest and DNA damage-inducing protein 45 (Gadd45) family members, including Gadd45a, Gadd45b, and Gadd45g, have been implicated in many basic processes, such as DNA repair [16], genome stability [17], epigenetic regulation [18], cell cycle arrest [19], apoptosis [20], tumor development [21], and embryogenesis [22]. Gadd45 proteins are small (~18 kD), with high homology. Gadd45a was the first member identified from Chinese hamster (CHO) cells [23]. Gadd45b was identified as a novel myeloid differentiation primary response gene induced by IL-6 [24]. Gadd45g was first described as an IL-2-inducible gene [25]. Gadd45g caught our most attention because depletion of Gadd45g cannot disrupt mESC maintenance [26], whereas Gadd45g is increased after Oct4 expression was knocked down [27–29]. In addition, Gadd45g was detected to be expressed in the scattered cells of the extraembryonic ectoderm and continues to increase after gastrulation during embryonic development [30]. Aside, the transcript of Gadd45g is induced in the E9.5 mouse embryos [31], and is important to the primary sex determination [31], testis development [31], initiation of neuronal differentiation [32], and lineage selection of hematopoietic stem cells [33]. Notably, Gadd45g is required for early embryonic cells to exit pluripotency and enter differentiation in Xenopus [34]. Based on these accumulated data, we speculated that Gadd45g may be a vital determiner for ESCs to initiate the differentiation process.

To validate this hypothesis, we used gain and loss of function methods to investigate the role of Gadd45g in the self-renewal and differentiation of ESCs. Overexpression of Gadd45g decreased cell proliferation and rapidly induced specification of endoderm and trophectoderm in mESCs. Gadd45g functions mainly via activation of MAPK signaling pathway. Importantly, GADD45G suppresses growth and EMT of human breast cancer cells in vivo and vitro and acts as an antitumor character.

## Results

### Enforced Gadd45g instructs mESCs exit from the undifferentiated state

**Fig. 1:**
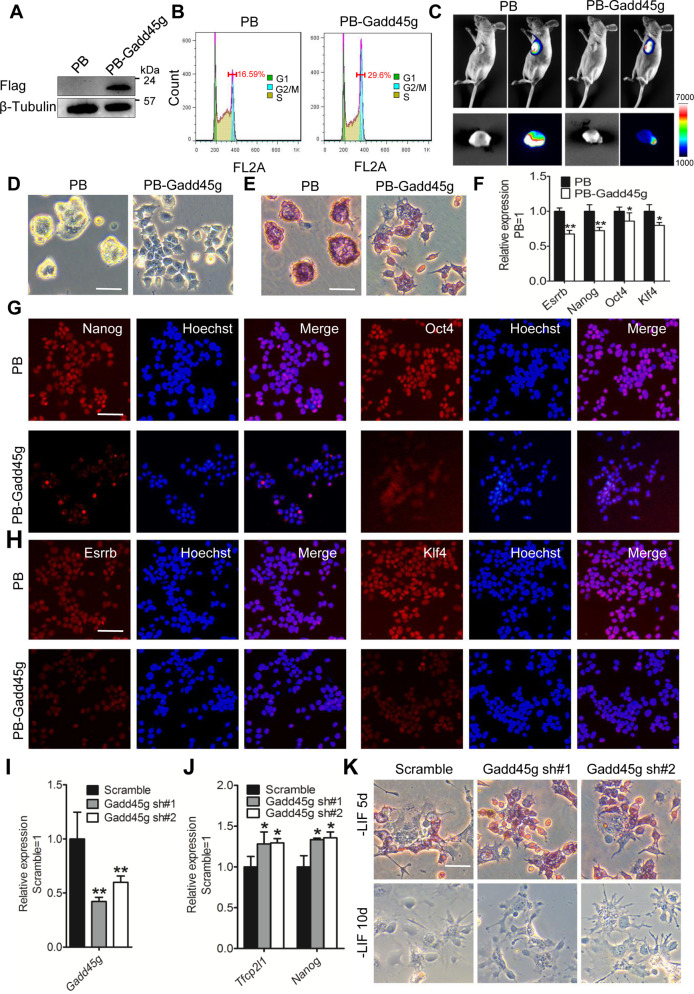
Overexpression of Gadd45g inhibits mESC self-renewal. **A** Western blot analysis of FLAG in 46 C mESCs overexpressing empty vector (PB) or FLAG-tagged mouse Gadd45g (PB-Gadd45g). β-tubulin was used as a loading control. **B** Flow cytometry was used to detect the changes of cell cycle distribution of PB and PB-Gadd45g 46 C mESCs. **C** Luciferase-tagged 46 C mESCs stably expressing PB or PB-Gadd45g were transplanted in nude mice. The tumors were imaged using Tanon-5200Multi system. **D,** **E** Morphology and Alkaline phosphatase (AP) staining of PB and PB-Gadd45g 46 C mESCs cultured in LIF/serum-containing medium for 2 passages. Bar, 100 μM. **F** Quantitative real-time PCR (qRT-PCR) analysis of the expression levels of self-renewal genes Esrrb, Nanog, Oct4, and Klf4. The data are represented as the means ± s.d. (*N* = 3 biological replicates). *p < 0.05, **p < 0.01 vs PB. **G,** **H** Immunofluorescence of the expression of pluripotency genes Oct4, Nanog, Esrrb, and Klf4 in PB and PB-Gadd45g 46 C mESCs. Bar, 100 μM. **I** qRT-PCR analysis of Gadd45g expression in 46 C mESCs infected with scramble or mouse Gadd45g shRNA lentiviruses (Gadd45g sh#1 and Gadd45g sh#2). The data are represented as the means ± s.d. (*N* = 3 biological replicates). **p < 0.01 vs scramble. **J** qRT-PCR analysis of self-renewal gene expression (Tfcp2l1 and Nanog) in scramble and Gadd45g shRNA mESCs. The data are represented as the means ± s.d. (*N* = 3 biological replicates). *p < 0.05 vs scramble. **K** AP staining of scramble and Gadd45g shRNA 46 C mESCs cultured in serum-containing medium without LIF for 5 days and 10 days. d, days. Bar, 100 μM.

To investigate the effect of Gadd45g on mESC maintenance, first, we generated a mESC line that overexpressed FLAG-tagged Gadd45g using a PiggyBac vector (PB-Gadd45g) and empty vector (PB) was used as control (Fig. 1A). After cultured in serum-containing medium in the presence of LIF, PB-Gadd45g mESCs were arrested in G2/M stage and exhibited slower proliferation rate than PB cells (Fig. 1B). To investigate whether Gadd45g overexpression has similar effects in vivo, luciferase-tagged 46 C cells expressing Gadd45g or PB vector were injected into nude mice. The luciferase signal indicated that teratomas was reduced in mice transplanted with the cells expressing Gadd45g compared with mice with the cells expressing PB (Fig. 1C). These results indicate that Gadd45g upregulation suppresses mESC proliferation.

In addition to a slow growth rate, PB-Gadd45g cells differentiated and exhibited low alkaline phosphatase (AP) activity after two passages, while PB cells maintained an undifferentiated morphology and sustained high AP activity (Fig. 1D, E). Overexpression of Gadd45g repressed the expression levels of pluripotency genes Esrrb, Nanog, Oct4, and Klf4 (Fig. 1F–H). These data collectively suggest that elevated expression of Gadd45g inhibits the self-renewal ability of mESCs.

As Gadd45g is a robust differentiation driver, we next wanted to decrease its transcription (Gadd45g sh#1 and Gadd45g sh#2). The expression levels of Gadd45g were decreased by ~40–60% compared with the scramble group (Fig. 1I). These cells were grown normally in LIF/serum condition. Subsequently, they were seeded in serum-containing medium without LIF. After 5 days, Gadd45g shRNA mESCs expressed higher levels of self-renewal markers (Tfcp2l1 and Nanog) and generated more AP-positive colonies than scramble cells (Fig. 1J, K). However, both of them became flat and differentiated after 10 days (Fig. 1K). Therefore, downregulation of Gadd45g can delay the differentiation of mESCs.

### Overexpression of Gadd45g induces specification of endoderm and trophectoderm in mESCs

**Fig. 2:**
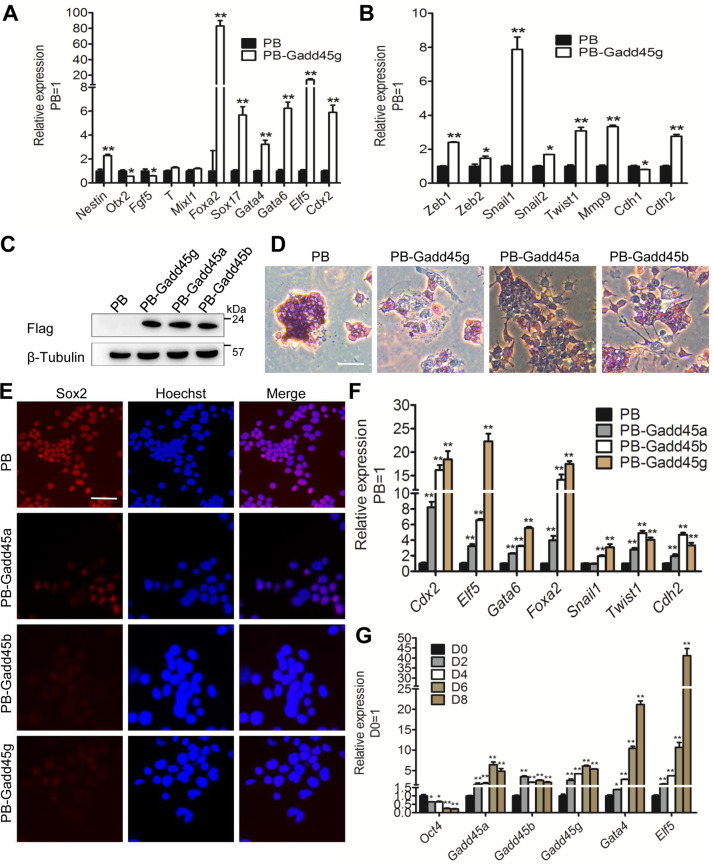
Overexpression of Gadd45 genes triggers lineage differentiation in mESCs. **A** qRT-PCR analysis of the expression of specific markers of different germ layers in PB and PB-Gadd45g 46 C mESCs. The data are represented as the means ± s.d. (*N* = 3 biological replicates). *p < 0.05, **p < 0.01 vs PB. **B** qRT-PCR analysis of the expression of epithelial-mesenchymal transition (EMT) marker genes in PB and PB-Gadd45g 46 C mESCs cultured in LIF/serum condition. The data are represented as the means ± s.d. (*N* = 3 biological replicates). *p < 0.05, **p < 0.01 vs PB. **C** Western blot analysis of FLAG in 46 C mESCs overexpressing FLAG-tagged mouse Gadd45 family genes (PB-Gadd45a, PB-Gadd45b, PB-Gadd45g) or PB. β-tubulin was used as a loading control. **D** AP staining of PB, PB-Gadd45a, PB-Gadd45b, and PB-Gadd45g 46 C mESCs cultured in LIF/serum-containing medium for 2 passages. Bar, 100 μM. **E** Immunofluorescence of pluripotency gene Sox2 in PB, PB-Gadd45a, PB-Gadd45b, and PB-Gadd45g 46 C mESCs. Bar, 100 μM. **F** qRT-PCR analysis of the expression of differentiation genes and EMT markers in PB, PB-Gadd45a, PB-Gadd45b, and PB-Gadd45g mESCs cultured in LIF/serum condition. The data are represented as the means ± s.d. (*N* = 3 biological replicates). **p < 0.01 vs PB. **G** qRT-PCR analysis of mouse Oct4, Gadd45a, Gadd45b, Gadd45g, Gata4, and Elf5 expression levels in 46 C mESCs-derived EBs collected from different days. The data are represented as the means ± s.d. (*N* = 3 biological replicates). *p < 0.05, **p < 0.01 vs D0. D0, Day 0.

Notably, Gadd45g-overexpressing mESCs quickly became flat, this promotes us to systematically investigate the Gadd45g-induced differentiated cells. As shown in Fig. 2A, PB-Gadd45g mESCs displayed higher levels of endoderm (Foxa2, Sox17, Gata4, and Gata6) and trophectoderm (Cdx2 and Elf5) markers than PB cells (Fig. 2A). However, there was no obvious changes in ectoderm (Otx2 and Fgf5) and mesoderm (T and Mixl1) markers (Fig. 2A), indicating that elevated expression of Gadd45g efficiently directs mESCs differentiation into endoderm and trophectoderm cells. Meanwhile, epithelial-mesenchymal transition (EMT) was induced with the emergence of differentiated cells, characterized by the increased levels of EMT associated markers (Zeb1, Zeb2, Snail1, Snail2, Twist1, Mmp9, and Cdh2) and the decreased level of mesenchymal-epithelial transition (MET) marker Cdh1 (Fig. 2B).

Because Gadd45g shares high homology with Gadd45a and Gadd45b, we further planned to investigate whether they have similar effects on mESC maintenance and differentiation. The FLAG-tagged three Gadd45 genes were transduced into 46 C mESCs, respectively (PB-Gadd45a, PB-Gadd45b, and PB-Gadd45g) (Fig. 2C). We observed that PB mESCs maintained an undifferentiated morphology and retained high AP activity grown in LIF/serum medium, while PB-Gadd45a, PB-Gadd45b, and PB-Gadd45g cells became flat and had low AP activity (Fig. 2D). Accordingly, Gadd45 gene overexpressing mESCs displayed lower level of the pluripotency gene Sox2 (Fig. 2E), whereas expressed higher levels of endoderm (Gata6 and Foxa2), trophectoderm (Cdx2 and Elf5), and EMT (Snail, Twist1, and Cdh2) markers than PB cells (Fig. 2F). These data suggest that all Gadd45 family genes can instruct mESCs exit from the self-renewal state.

To examine the expression patterns of Gadd45 members during mESC differentiation, 46 C mESCs were cultured in suspension to form embryoid bodies (EBs) where differentiation proceeds into the three germ layers. The expression of Oct4 was gradually decreased, while the expression levels of Gadd45 family genes and differentiation genes Gata4 and Elf5 were increased at mRNA and protein levels (Figs. 2G and S1), suggesting that Gadd45 family members are closely related to the initiation of mESC differentiation.

To examine whether Gadd45 genes have cross compensatory functionality in mESCs, we designed two shRNAs (sh#1 and sh#2) for each Gadd45 gene to inactive their expression. Stable knockdown of Gadd45 transcript levels was observed (Figure S2A–C). Then, we selected Gadd45a, Gadd45b, and Gadd45g sh#1 lentiviruses, which have the best gene interference effects, to infect 46 C mESCs and established Gadd45a/b/g triple knockdown cell line (Fig. S2D). When cultured in serum-containing medium without LIF for 5 days, Gadd45a/b/g triple knockdown cells generated more AP-positive colonies and expressed higher levels of Tfcp2l1, Nanog, and Oct4 than single Gadd45 gene shRNA cells (Fig. S2E and F). However, all of them differentiated after 10 days (Fig. S2E). These data indicate that there is considerable redundancy among the functions of Gadd45 genes.

### Gadd45g activates MAPK signaling pathway

**Fig. 3:**
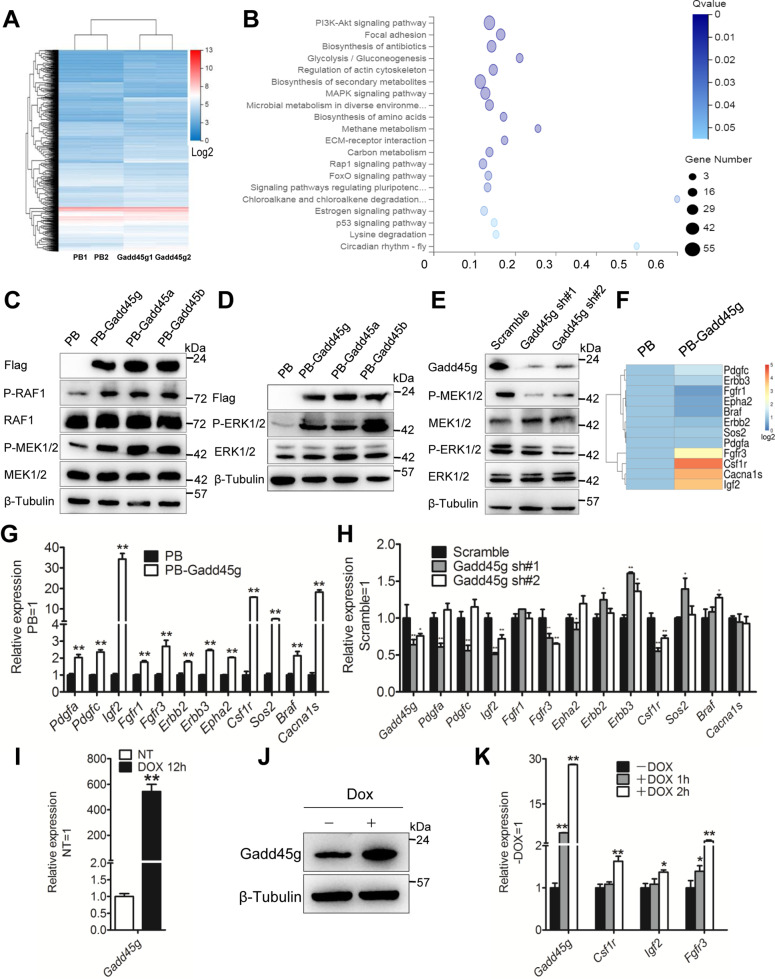
Gadd45g promotes mESC differentiation through activation of MAPK signaling pathway. **A** Heatmap showing the expression patterns of PB and PB-Gadd45g 46 C mESCs. Genes were ranked according to the level of log2-fold change. **B** The KEGG analysis of the DEGs regulated by Gadd45g. **C** Western blot analysis of FLAG, RAF1, P-RAF1, MEK1/2, and P-MEK1/2 in 46 C mESCs overexpressed Gadd45 genes or PB. β-tubulin was used as a loading control. **D** Western blot analysis of FLAG, ERK1/2, and P-ERK1/2 levels in Gadd45 genes overexpressing 46 C mESCs. β-tubulin was used as a loading control. **E** Western blot analysis of Gadd45g, MEK1/2, P-MEK1/2, ERK1/2, and P-ERK1/2 levels in scramble and Gadd45g shRNA 46 C mESCs cultured in LIF/serum medium. β-tubulin was used as a loading control. **F** Heatmap showing the DEGs upregulated by Gadd45g and associated with the MAPK signaling pathway. Genes were ranked according to the level of log2-fold change. **G** qRT-PCR analysis of the expression of candidates showed in panel **F**. The data are represented as the means ± s.d. (*N* = 3 biological replicates). **p < 0.01 vs PB. **H** qRT-PCR analysis of the expression of the indicated genes (showed in panel **F**) and Gadd45g in the scramble and Gadd45g shRNA 46 C mESCs cultured in LIF/serum medium. The data are represented as the means ± s.d. (*N* = 3 biological replicates). *p < 0.05, **p < 0.01 vs scramble. **I** qRT-PCR analysis of Gadd45g expression level in i-Gadd45g mESCs treated with or without Dox for 12 h. The data are represented as the means ± s.d. (*N* = 3 biological replicates). **p < 0.01 vs NT. NT no treatment. **J** Western blot analysis of Gadd45g levels in i-Gadd45g ESCs cultured in the presence or absence of 2 μg Dox. β-tubulin was used as a loading control. **K** qRT-PCR analysis of expression levels of Gadd45g, Csf1r, Igf2, and Fgfr3 in i-Gadd45g ESCs treated with Dox for 1 or 2 h. The data are represented as the means ± s.d. (*N* = 3 biological replicates). *p < 0.05, **p < 0.01 vs −DOX.

Compared with Gadd45a and Gadd45b, Gadd45g induced higher levels of differentiation-associated genes when overexpressed but expressed higher levels of self-renewal genes after knockdown (Figs. 2D–F and S2F), we next focused on Gadd45g to understand the mechanism by which Gadd45 gene overexpression suppresses mESC self-renewal. First, high-throughput sequencing was performed to analyze the differently expression genes (DEGs) regulated by PB and PB-Gadd45g (GEO Number: GSE172474). Compared with PB, PB-Gadd45g induced 799 upregulated genes and 468 downregulated genes by two folds or greater (Fig. 3A). Second, to gain an insight into how overexpression of Gadd45g induced differentiation, we analyzed the DEGs by KEGG method and found that many candidates are enriched in PI3K/AKT and MAPK signaling pathways (Fig. 3B). MAPK singling is important for mESCs to initiate differentiation [35], we therefore wanted to investigate whether Gadd45g overexpression was engaged in the activation of this pathway. Western blot was carried out to examine the levels of total and phosphorylated RAF1, MEK1/2, and ERK1/2 proteins (p-RAF1, p-MEK1/2, and p-ERK1/2), all of them are the key components of MAPK signaling pathway. Overexpression of Gadd45 genes had no effects on the total protein levels of RAF1, MEK1/2, and ERK1/2, but was able to increase their phosphorylation levels (Fig. 3C, D). In contrast, knockdown of Gadd45g obviously reduced the phosphorylation of MEK1/2 and ERK1/2 (Fig. 3E). These data indicate that Gadd45g positively mediates the activation of MAPK signaling pathway.

To clarify how Gadd45g stimulates MAPK pathway, we further used qRT-PCR to validate the expression of those 12 candidate genes induced by Gadd45g (Fig. S3), including Pdgfa, Pdgfc, Igf2, Fgfr1, Fgfr3, Erbb2, Erbb3, Epha2, Csf1r, Sos2, Braf, and Cacna1s (Fig. 3F and G). At the same time, we also evaluated the expression of these genes in Gadd45a- and Gadd45b-overexpressing mESCs and observed that most of them are significantly induced (Fig. S4). Next, to test whether we can observe opposite expression patterns after Gadd45g was downregulated, we detected the transcripts of these candidates in Gadd45g shRNA cells. Only the transcripts of Igf2, Csf1r, and Fgfr3 are decreased in both two Gadd45g shRNA cell lines (Fig. 3H). To determine whether their transcription is sensitive to Gadd45g upregulation, we used an inducible cassette exchange (ICE) system to generate a mESC line carrying a doxycycline (Dox) inducible Gadd45g transgene (i-Gadd45g) [36]. Dox treatment could effectively induce the expression of Gadd45g in i-Gadd45g mESCs (Fig. 3I, J). Subsequently, Dox was supplemented into medium for a short-time treatment and the transcripts of Igf2, Csf1r, and Fgfr3 were increased (Fig. 3K), suggesting that the transcripts of Igf2, Csf1r, and Fgfr3 can quickly be induced in response to Gadd45g upregulation.

### Inhibition of MAPK signaling pathway eliminates the lineage commitment induced by Gadd45g

**Fig. 4:**
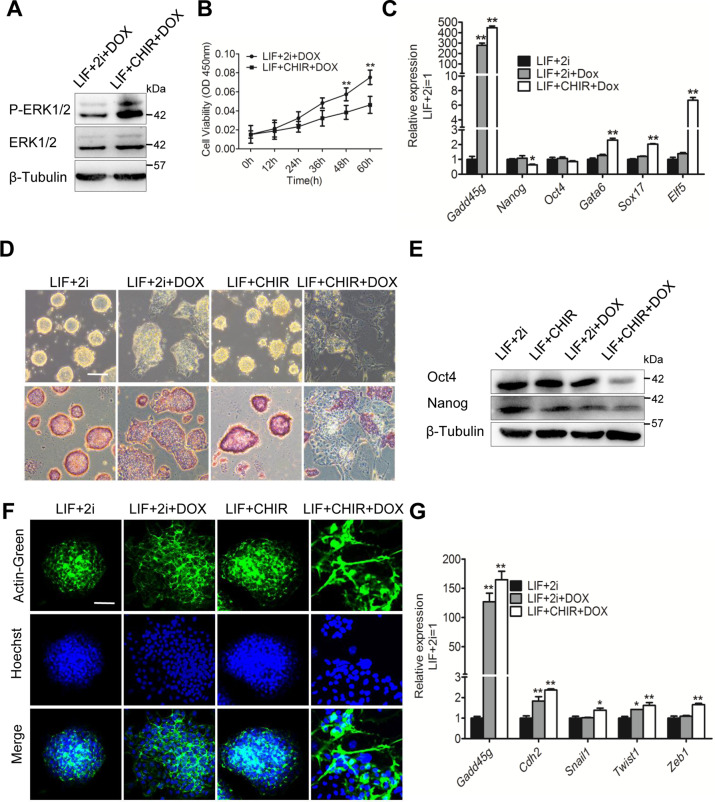
Suppression of the MAPK signaling pathway inhibits differentiation caused by Gadd45g overexpression. **A** Western blot analysis of ERK1/2 and P-ERK1/2 levels in i-Gadd45g ESCs cultured in LIF/CHIR/Dox condition in the presence or absence of PD03. β-tubulin was used as a loading control. **B** The CCK8 assay was performed to analyze the proliferation of i-Gadd45g ESCs cultured in LIF/CHIR/Dox condition in the presence or absence of PD03. The data are represented as the means ± s.d. (*N* = 3 independent biological experiments). **p < 0.01 vs LIF + CHIR + Dox. **C** qRT-PCR analysis of Gadd45g, self-renewal (Oct4 and Nanog) and differentiation (Gata6, Sox17, and Elf5) gene expression levels in i-Gadd45g ESCs cultured in the indicated conditions. The data are represented as the means ± s.d. (*N* = 3 biological replicates). *p < 0.05, **p < 0.01 vs LIF + 2i. **D** Morphology and alkaline phosphatase (AP) staining of i-Gadd45g ESCs cultured in the indicated conditions. Bar, 100 μM. **E** Western blot analysis of Oct4 and Nanog protein levels in i-Gadd45g ESCs cultured in the indicated conditions. β-tubulin was used as a loading control. **F** Actin-Tracker Green-488 fluorescent probe staining of i-Gadd45g ESCs cultured in the indicated conditions. Bar, 100 μM. **G** qRT-PCR analysis of the expression levels of Gadd45g and EMT marker genes in i-Gadd45g ESCs cultured in the indicated conditions. The data are represented as the means ± s.d. (*N* = 3 biological replicates). *p < 0.05, **p < 0.01 vs LIF + 2i.

We further wanted to examine whether blocking this pathway with PD03, a specific MEK inhibitor, can inhibit the phenotype mediated by Gadd45g. As expected, addition of PD03 could efficiently decrease the p-ERK1/2 level in i-Gadd45g mESCs treated with Dox (Fig. 4A), and sufficiently restored the proliferation suppressed by Dox (Fig. 4B). Moreover, i-Gadd45g cells, maintained in LIF/2i condition, expressed higher levels of Oct4 and Nanog and retained stronger AP activity, but displayed lower levels of endoderm and trophectoderm genes (Gata6, Sox17, and Elf5) than LIF/CHIR-treated cells in the presence of Dox (Fig. 4C–E), meaning that Gadd45g upregulation fails to induce differentiation in the presence of PD03 (Fig. 4C–E). The similar results could also be observed in 46 C mESCs (Fig. S5A and B). Notably, downregulation of Gadd45g was not sufficient to replace the function of PD03 to maintain the undifferentiated state together with CHIR (Fig. S6).

To confirm that PD03 was capable of inhibiting the differentiation-associated EMT phenotype, we checked the changing of cell cytoskeleton with Actin-Tracker Green-488 fluorescent probe and found that the cytoskeleton became stretched and dispersed after Dox was added, whereas addition of PD03 was able to block the EMT induced by Dox (Fig. 4F). Besides, addition of PD03 significantly decreased the expression levels of EMT markers induced by Dox (Fig. 4G). Similar phenotype was observed in 46 C mESCs (Fig. S5C and D). Overall, these results indicate that reduced activity of MAPK pathway is sufficient to repress the differentiation caused by Gadd45g overexpression.

### Upregulation of GADD45G inhibits the proliferation of breast cancer cells

**Fig. 5:**
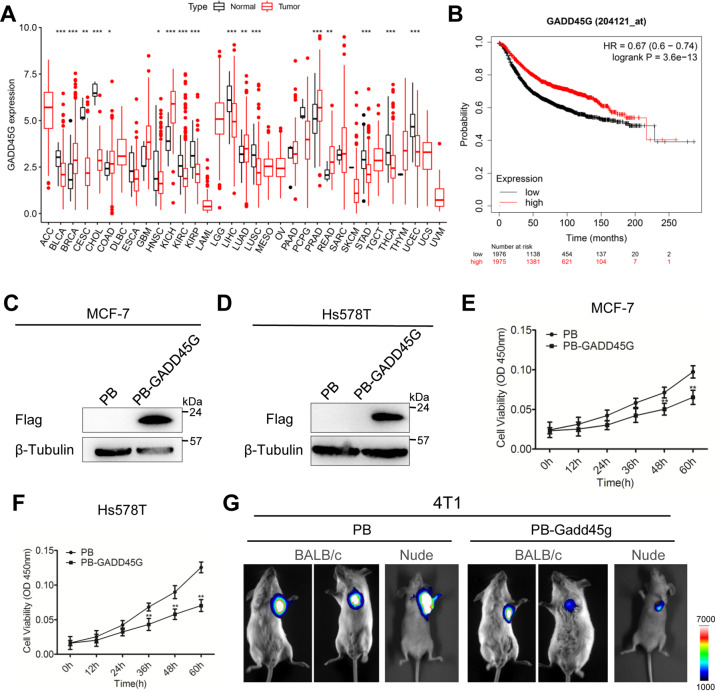
Overexpression of GADD45G limits the propagation of breast cancer cells. **A** Analysis of GADD45G expression in different kinds of normal and cancer samples from TCGA database. **B** The expression level of GADD45G is related to the overall survival time of breast cancer patients. **C**, **D** Western blot analysis of FLAG in MCF7 and Hs578T cells overexpressing FLAG-tagged human GADD45G (PB-GADD45G) or empty vector. β-tubulin was used as a loading control. **E**, **F** CCK8 assay was performed to assay the effects of GADD45G on the proliferation of MCF7 and Hs578T cells. The data are represented as the means ± s.d. (*N* = 3 independent biological experiments). **p < 0.01. **G** Luciferase-tagged mouse breast cancer cell line 4T1 stably expressing PB or PB-Gadd45g were transplanted in BALB/c and nude mice. Luciferase signals were captured by Tanon-5200Multi system.

Human induced pluripotent stem cells (iPSCs) have distinguished features with mESCs, whereas overexpression of GADD45G also could lead to human iPSC differentiation (Fig. S7A and B). Notably, in the development of many different kinds of cancers, GADD45G is lowly expressed and is considered to be a functional tumor suppressor (Fig. 5A) [37–39], which is similar to the result we observed in mESCs (Fig. 1B and C). We selected breast cancer cells to do further investigation, as the TCGA database analysis showed that GADD45G is highly expressed in breast cancer patients (Fig. 5A), implying that GADD45G may be an oncogene. However, the survival curve showed that high level of GADD45G is benefit for the overall survival rate of patients (Fig. 5B). These contradictory results promote us to modulate the transcript of GADD45G to make sure its specific function. FLAG-tagged human GADD45G (PB-GADD45G) and PB were transduced into breast cancer cell lines MCF7 and Hs578T, respectively (Fig. 5C, D). There is no obvious difference in apoptosis between PB and PB-GADD45G breast cancer cells (Fig. S8). The growth of GADD45G-overexpressing MCF7 and Hs578T cells was slower than that of PB cells (Fig. 5E, F). To confirm the proliferation-inhibiting activity of GADD45G in vivo, Luciferase-tagged 4T1, a mouse breast cancer cell line, expressing Gadd45g were established. After transplanted under the breast pads of BALB/c and nude mice, both PB and PB-Gadd45g cells could generate tumors in mice, while the tumor volumes were reduced derived from Gadd45g cells (Fig. 5G). These data demonstrated that GADD45G overexpression decreases breast cancer cell proliferation.

### GADD45G suppresses breast cancer metastasis

**Fig. 6:**
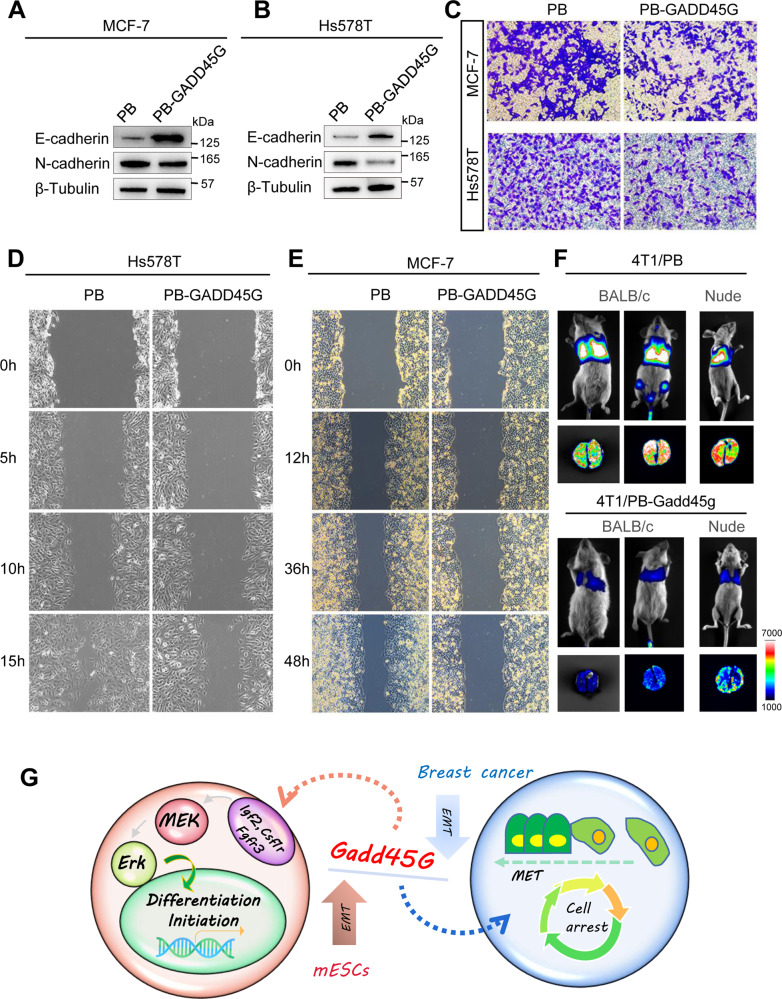
Overexpression of GADD45G decreases the migration of breast cancer cells. **A,B** Western blot analysis of E-cadherin and N-cadherin levels in MCF7 or Hs578T cells overexpressing PB or PB-GADD45G. β-tubulin was used as a loading control. **C** Transwell experiments were used to analyze the migration ability of MCF7 and Hs578T cells overexpressing PB or PB-GADD45G. **D,** **E** The scratch experiments were used to evaluate the migration abilities of MCF7 or Hs578T cells overexpressing PB or PB-GADD45G. **F** Luciferase-tagged mouse breast cancer 4T1 cells stably expressing PB or PB-Gadd45g were transplanted in mice. Metastasis was imaged using Tanon-5200Multi system. **G** Schematic diagram of the role of Gadd45g in mouse ESCs and breast cancer cells. Overexpression of Gadd45g triggers ESC differentiation via activation of MAPK signaling, and suppresses the proliferation and invasion of breast cancer cells in vivo and in vitro.

To measure the metastasis-regulating activity of GADD45G, we examined the migration and invasion events with three approaches. First, western blot was performed to detect the levels of EMT makers. As shown in Fig. 6A, B, PB-GADD45G MCF7 and Hs578T cells expressed higher levels of MET maker E-cadherin but exhibited lower level of EMT gene N-cadherin (Fig. 6A, B). Second, transwell assessments showed that MCF7 and Hs578T cells overexpressing GADD45G exhibited decreased invasion capabilities when compared with PB cells (Fig. 6C). Similarly, scratch experiments were used to evaluate the migration of cells, and the results demonstrated that GADD45G had an inhibitory effect on breast cancer migration (Fig. 6D, E). Finally, Luciferase-tagged 4T1 cells expressing PB or PB-Gadd45g were also transplanted into BALB/c and nude mice via tail vein. Mice injected with PB cells developed more lung metastasis than mice injected with PB-Gadd45g cells (Fig. 6F). These data demonstrate that GADD45G expression suppresses the metastases of breast cancer cells.

## Discussion

The self-renewal markers of ESCs often promote cancer cell proliferation and metastasis. However, the research on ESC differentiation initiation and the effects of differentiation-associated genes on cell carcinogenesis are relatively rare. Our project reveal that Gadd45g upregulation can instruct ESCs exit from undifferentiated state and enter into endoderm and trophectoderm commitment. The MAPK singling pathway is largely responsible for the effect of Gadd45g in mESCs. In addition, GADD45G plays as an antitumor gene and represses proliferation and migration of breast cancer cells in vivo and in vitro (Fig. 6G).

There are several previous reports supporting our findings that Gadd45 family genes are differentiation initiation associated candidates. In situ hybridizations of mouse embryos reveals that Gadd45g is strongly expressed in neuron precursors, Gadd45a expression is enriched in the tip of the closing neural tube and Gadd45b is expressed highly in the chorion [30]. Functional studies reveals that overexpression of Gadd45g not only induces neuronal differentiation of P19 cells [32], but is also critical to Xenopus and medaka fish embryos exit from pluripotency and enter neural development progress [34, 40, 41]. These data indicate that the role of Gadd45g in the early neuro-directed differentiation of different species is conserved. However, overexpression of Gadd45g induced endoderm and trophectoderm lineage cells in mESCs (Figs. 1D, E and 2A); that may be due to the presence of serum, which can completely block the neural differentiation of mESCs [42]. In addition, Gadd45a/b/g triple gene knockout mESCs can be established in vitro [26]. Knockdown of Gadd45g could delay differentiation (Fig. 1J, K). Additionally, the transcript level of Gadd45g was increased upon mESC differentiation (Fig. 2G and S1) [26, 27], meaning that Gadd45g is dispensable for mESC maintenance but associated with the differentiation initiation. Moreover, the transcript of Gadd45g is essential for the normal progress of EMT occur in rabbit early embryos [43]. The similar effect of Gadd45 genes on EMT transition can be observed in mESCs (Fig. 2B, F). EMT transition are closely with the ESC differentiation [44–47]. Inhibition of EMT favors the self-renewal of ESCs [47, 48]. Finally, we found that upregulation of Gadd45g activates MAPK signaling pathway (Fig. 3C, D), which is essential for the spontaneous differentiation of mESCs [35]. In contrast to mESCs, human iPSCs and ESCs rely on MAPK signaling to sustain stemness [49]. Interestingly, upregulation of GADD45G induced human iPSC differentiation (Fig. S7B), implying that there are other mechanisms downstream of GADD45G to negatively regulate the pluripotency of human pluripotent stem cells, like DNA demethylation [26].

Another point is that GADD45G significantly inhibits the migration and invasion of breast cancer cells (Fig. 6C–F). Actually, the antitumor activity of GADD45G has been investigated before. Its transcriptional downregulation was frequently found in many tumor cells, including Hodgkin’s lymphoma, nasopharyngeal carcinoma, cervical carcinoma, esophageal carcinoma, and lung carcinoma [39]. The decreased levels may be due to the hypermethylation of the proximal promoter of GADD45G [39, 50]. Ectopic expression of GADD45G not only robustly elicits cellular senescence evasion [37], but also interacts with E-cadherin to enhance its membrane level [51]. We also observed increased E-cadherin protein in response to GADD45G upregulation (Fig. 6A). Additionally, overexpression of GADD45G has ability to restrict proliferation by accumulation of cells arrested in the different stages of cell cycle through association with p21 and cyclin B [52–56]. Due to high homology of GADD45 genes, GADD45A and GADD45G exert similar effects on the development of cancers [54, 57, 58]. In this study, we demonstrated that Gadd45g could activate MAPK pathway (Fig. 3B–E). High ERK protein expression levels have been shown to correlate with shorter survival in Triple-Negative Breast Cancer Patients [59, 60], implying that there are other mechanisms excepted MAPK signaling responsible for effect of GADD45G on the development of breast cancer. However, whether GADD45 family genes share a common set of mechanisms to inhibit the migration and invasion of different cancer cells, more experiments are required to be performed.

In summary, our study demonstrates that Gadd45 genes are pivotal factors involved in the lineage commitment of ESCs. We further demonstrated that MAPK pathway is a major signaling responding to Gadd45g upregulation to direct mESCs exit from self-renewal and enter into the endoderm and trophectoderm cell fates. Meanwhile, GADD45G plays a critical role in inhibiting breast cancer cell migration and invasion. Our research not only demonstrated Gadd45g as a pivotal differentiation inducer of ESCs, but also will help people explore more drugs to better control cell differentiation and cancer treatment.

## Materials and methods

### Cell culture

46 C mESCs, kindly provide by professor Qi-Long Ying (University of Southern California), were seeded on 0.1% gelatin-coated cell culture plates at 37 °C in an incubator supplemented with 5% carbon dioxide. The composition of the mESC basic medium is DMEM (2122149, Biological Industries, Israel) supplemented with 10% FBS (FND500, ExCell Bio, Australia), 1× MEM nonessential amino acids (N1250, Solarbio, China), 0.1 mM β-mercaptoethanol (M3148, Sigma), and 1000 U/ml LIF (LIF1010, Millipore, USA). i-Gadd45g mESCs were maintained in basic medium supplemented with PD0325901 (1 μM, HY-10254, MedChemExpress) and CHIR99021 (3 μM, HY-10182, MedChemExpress). Human transgenes-free induced pluripotent stem cells were kindly provided by NuwaCell.Ltd, China (ZSSY-001) and were cultured in ncTarget medium (RP01020, NuwaCell.Ltd, China). MCF7 and Hs578T cells were purchased from the National Collection of Authenticated Cell Cultures (Chinese Academy of Sciences) and were cultured in DMEM medium supplemented with 10% FBS.

### Plasmid construction

The coding regions of mouse and human Gadd45g were amplified by PCR and were inserted into PggyBac transposon vectors. For RNA interference (RNAi) in mESCs, small hairpin RNA (shRNA) constructs were designed to target gene-specific regions of Gadd45a, Gadd45b, and Gadd45g and then were cloned into pLKO.1-TRC (#10878, Addgene). The primer sequences used have been listed in Tables S1 and S2.

### Alkaline phosphatase (AP) activity assay

The cells were fixed in 4% paraformaldehyde at room temperature for 2 min, after washed twice with PBS, cells were incubated in AP staining reagent (C3206, Beyotime Biotechnology, China) at room temperature for 30 min in the dark. After washing twice with PBS, we observed and took pictures under the Leica DMI8 microscope.

### Western blot

Cells were lysed in ice-cold RIPA cell buffer (P0013B, Beyotime Biotechnology, China) supplemented with protease inhibitors cocktail. The protein samples were then separated on a 10%- or 15%-PAGE gel and electrotransferred to a PVDF membrane. After blocking, the membrane was incubated with specific primary antibodies overnight at 4 °C and followed by a HRP-conjugated secondary antibody at room temperature. Images were captured under the Chemiluminescence Gel Imaging System Tanon-5200Multi (Shanghai Tianneng, China). The primary antibodies are FLAG (SG110-26, GNI, Japan, 1:1000), Nanog (14295-1-AP, Proteintech, USA, 1:1000), Gadd45g (SC-33173, Santa Cruz, USA, 1:500), Gadd45a (UPA06635, Gene Universal, China, 1:500), Gadd45b (UPA01987, Gene Universal, China, 1:500), MEK1/2 (380797, ZENBIO, China, 1:1000), Phospho-MEK1/2 (Ser217/221) (310050, ZENBIO, China, 1:1000), ERK1/2 (201245-4A4, ZENBIO, China, 1:500), Phospho-ERK1 (Thr202/Tyr204)/ERK2 (Thr185/Tyr187) (301245, ZENBIO, China, 1:500), Raf1 (251817, ZENBIO, China, 1:1000), Phospho- Raf1 (Ser338) (D155090, BBI, China, 1:1000), β-tubulin (200608, ZENBIO, China, 1:2000), E-cadherin (201283, ZENBIO, China, 1:1000), and N-cadherin (383341, ZENBIO, China, 1:1000).

### Quantitative real-time PCR (qRT-PCR)

According to the manufacturer’s protocol, the TRIzol Up Plus RNA Kit (R0027, Beyotime, China) was used to isolate total RNA from cells. cDNA was synthesized from 1 µg of total RNA with the Hifair III 1st Strand cDNA Synthesis SuperMix for qPCR (11141ES60, YEASEN, China). mRNA expression levels were determined using the Hieff qPCR SYBR Green Master Mix (11201ES08, YEASEN, China) in a PikoReal Real-Time PCR machine (Thermo Scientific, USA). The relative expression level was determined by the 2-ΔCq method and normalized to mouse RPL19 expression. The primers used are listed in Table S3.

### Immunofluorescence staining

The cells were fixed in 4% paraformaldehyde for 30 min and then were washed twice with PBS. After incubated for 2–3 h in blocking buffer containing 5% BSA and 0.2% Triton X-100, the cells were probed with the primary antibodies overnight at 4 °C. After washed three times with PBS, cells were incubated in blocking buffer containing a specific fluorescent secondary antibody and Hoechst 33342 (H3570, Invitrogen, 1:10,000) at 37 °C for 1 h. The cells were photographed under a Leica DMI8 microscope. The primary antibodies are Oct4 (SC-5279, Santa Cruz, USA, 1:500), Nanog (14295-1-AP, Proteintech, 1:500), Klf4(381633, ZENBIO, China, 1:500), and Sox2 (66411-1-Ig, Proteintech, 1:500).

### Cell transfection and infection

For gene overexpression, cells were transfected with 2 μg PiggyBac along with 2 μg transposase vector using Hieff Trans^TM^ Liposomal Transfection Reagent (40802ES03, YEASEN, China) according to the manufacturer’s instruction. For knockdown experiments, pLKO.1-TRC-based lentiviral vectors and packaging plasmids pMD2.G and psPAX2 were co-transfected into 293FT cells. The virus supernatant was collected after 48 h of transfection and was filtered with a 0.45 μm filter membrane. Then the viral supernatant was added into the culture medium to infect cells. After 48 h, cells were selected by adding Puromycin, Blasticidin, or Hygromycin.

### Embryoid bodies (EB) differentiation

In order to verify the relationship between Gadd45 genes and mESC differentiation, 5 × 10^6^ mESCs were cultured in 20% FBS in 10 cm petri dish to form embryoid bodies (EBs), which were collected and lysed in TRIzol or RIPA every two days.

### Transwell assay

The cells were digested by trypsin into single cells and then were counted by Countess 3 (Invitrogen). Six thousands cells were resuspended in DMEM without FBS and then were put into the transwell upper chamber. Next, the normal serum-containing medium was put into the 24-well plate below the chamber. After incubation for 24–48 h, the nonmigrated cells were erased by a cotton swab. The migrated cells were fixed with methanol and were stained with 0.1% crystal violet. The images were taken with a Leica DMI8 microscope.

### Flow cytometry

The cells were collected, centrifuged, and washed with precooled PBS for three times. Subsequently, 70% precooled ethanol was used to fix the cells for 12–24 h. After the ethanol was discarded, cells were stained with the reagents of the cell cycle and apoptosis detection kit (C1052, Beyotime Biotechnology, China) and were incubated at 37 °C for 30 min in the dark. All samples were put on ice and were analyzed by flow cytometry instrument (BD bioscience) to detect the cell cycle changes.

### CCK8 assay

Two thousands to five thousands cells were seeded in a 96-well plate at 37 °C. Cell Counting Kit-8 (CCK8) reagent was added every 12 h and cells were incubated for 1 h in the dark at 37 °C. The absorbance was measured at 450 nm in a microplate reader (Molecular Devices).

### Animal models

Four- to five-week-old BALB/c or BALB/c-nude female mice were used in our experiments. For in vivo imaging technology, 2–5 × 10^6^ cells carrying Luciferase (LUC) coding sequences were injected into mice. After 10–15 days, D-Luciferin potassium salt (150 mg/kg) (40902ES02, YEASEN, China) was injected into the abdominal cavity of anesthetized mice, the latter were placed in the Chemiluminescence Gel Imaging System Tanon-5200Multi (Shanghai Tanon, China) to observe live imaging.

### Accession number

Our Microarray dataset has been deposited in the GEO database under ID number GSE172474.

### Statistical analysis

Mice with poor physical condition were excluded before grouping. Quantitative data are representative of at least three biological replicates or three independent experiments. All data are reported as the mean ± s.d., Student’s *t*-test is used to determine the significance of the comparison difference by using GraphPad Prism 8 software. Values with *p* < 0.05 are considered statistically significant. No animal randomization was used. No blinding was used. No statistical method was used to predetermine the sample size.

## Supplementary information

The expression levels of Gadd45 genes in EB cells

Knockdown of Gadd45 family genes delay mESC differentiation

The MAPK signal pathway diagram

Gadd45a and Gadd45b induce the expression of MAPK signaling pathway associated genes

Effects of PD03 on 46C mESCs overexpressing Gadd45g

CHIR fails to maintain stemness in Gadd45g shRNA mESCs

. GADD45G overexpression promotes human iPSC differentiation

The apoptosis of PB and PB-GADD45G breast cancer cells

List of primers for amplifying Gadd45 members

List of sequences used for gene knockdown

List of primers used for qRT-PCR analysis

Supplementary figure legends

## Data Availability

All the data generated or analyzed during this this study are included in this published article.
