# Supplementary figures and images for: Gadd45g initiates embryonic stem cell differentiation and inhibits breast cell carcinogenesis
Source: Cell Death Discov. 2021 Oct 2;7:271. doi: 10.1038/s41420-021-00667-x (PMC8487429; doi:10.1038/s41420-021-00667-x)

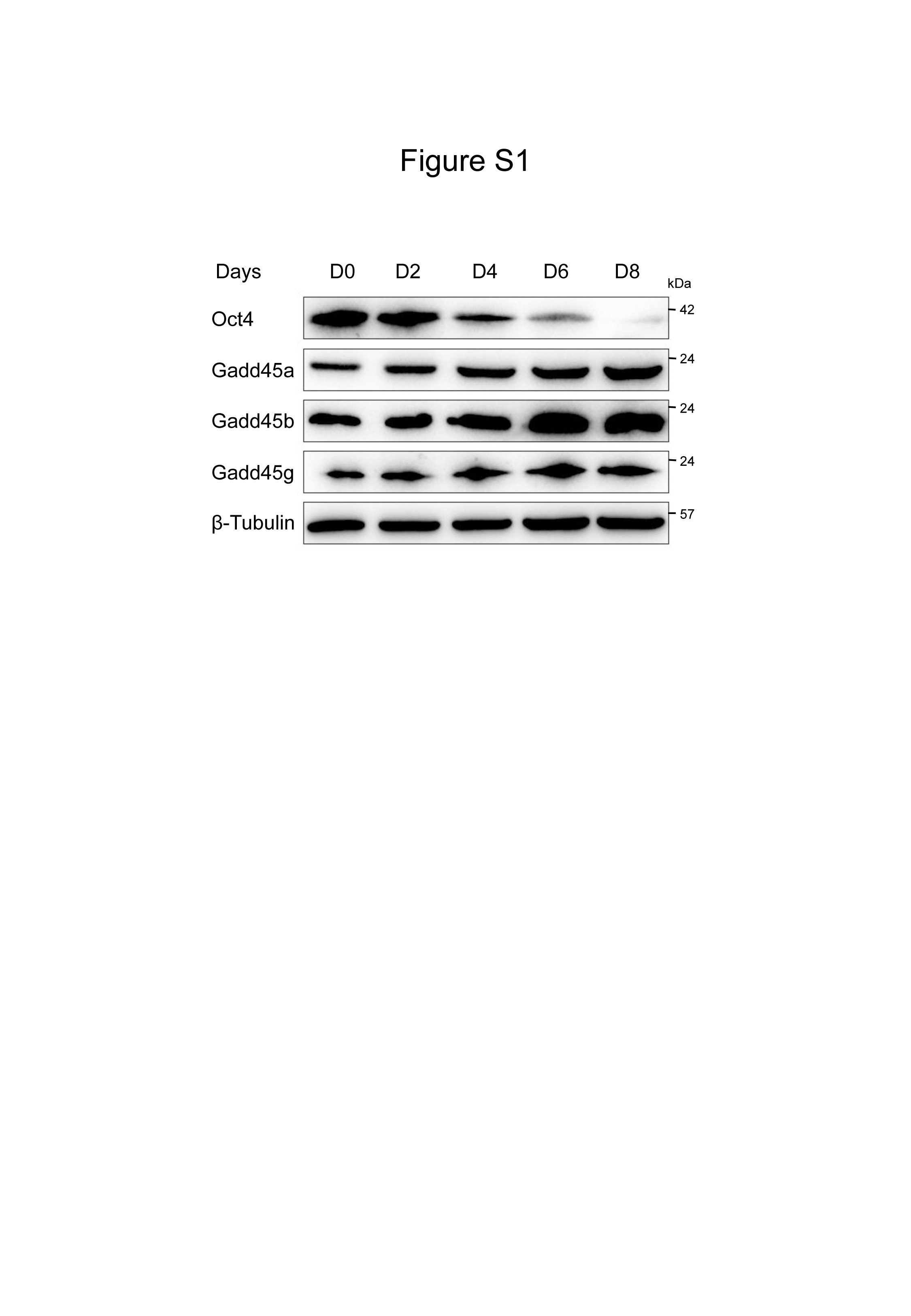

Supplement: Supplementary file 1 — The expression levels of Gadd45 genes in EB cells [file 41420_2021_667_MOESM1_ESM.tif]

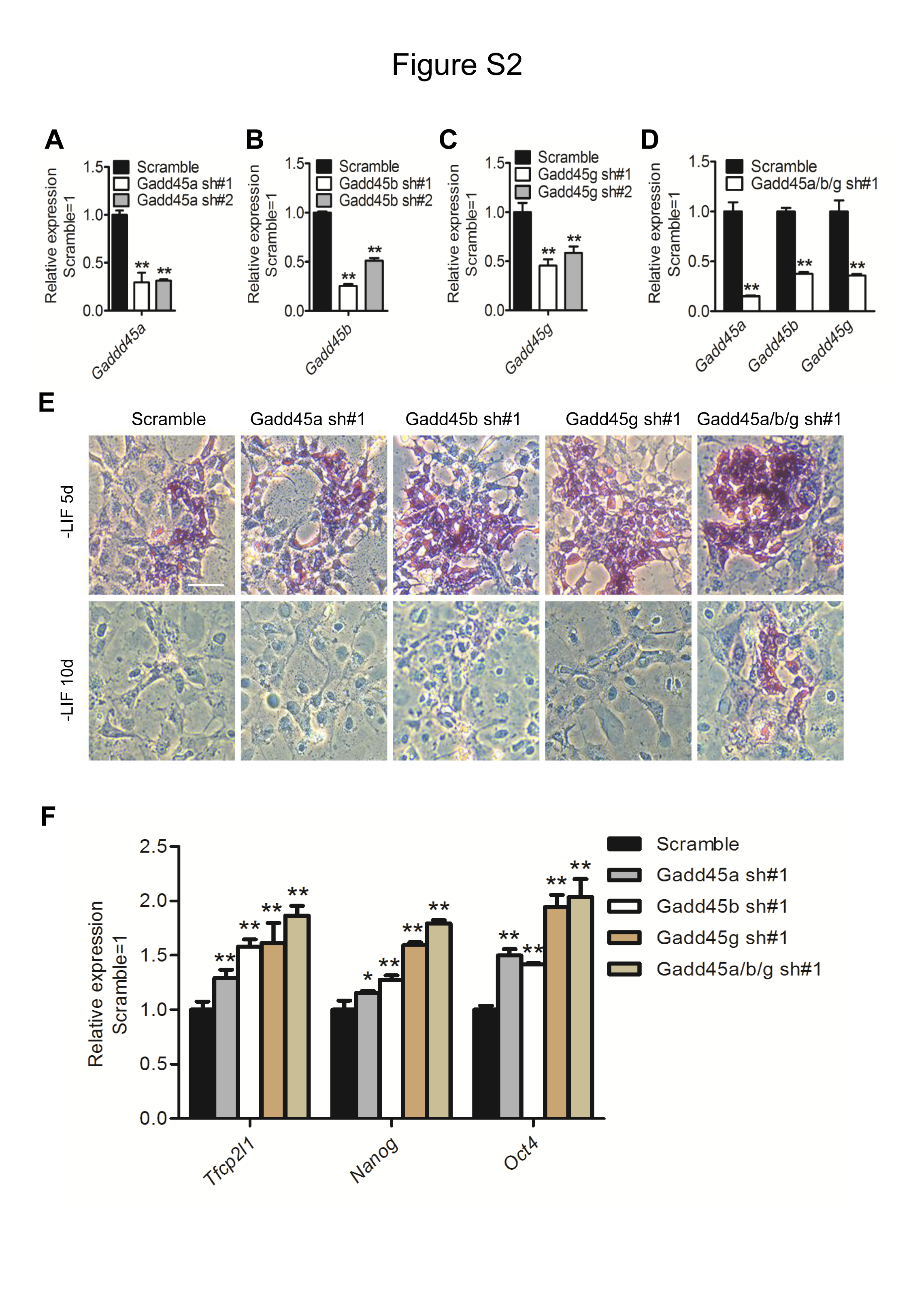

Supplement: Supplementary file 2 — Knockdown of Gadd45 family genes delay mESC differentiation [file 41420_2021_667_MOESM2_ESM.tif]

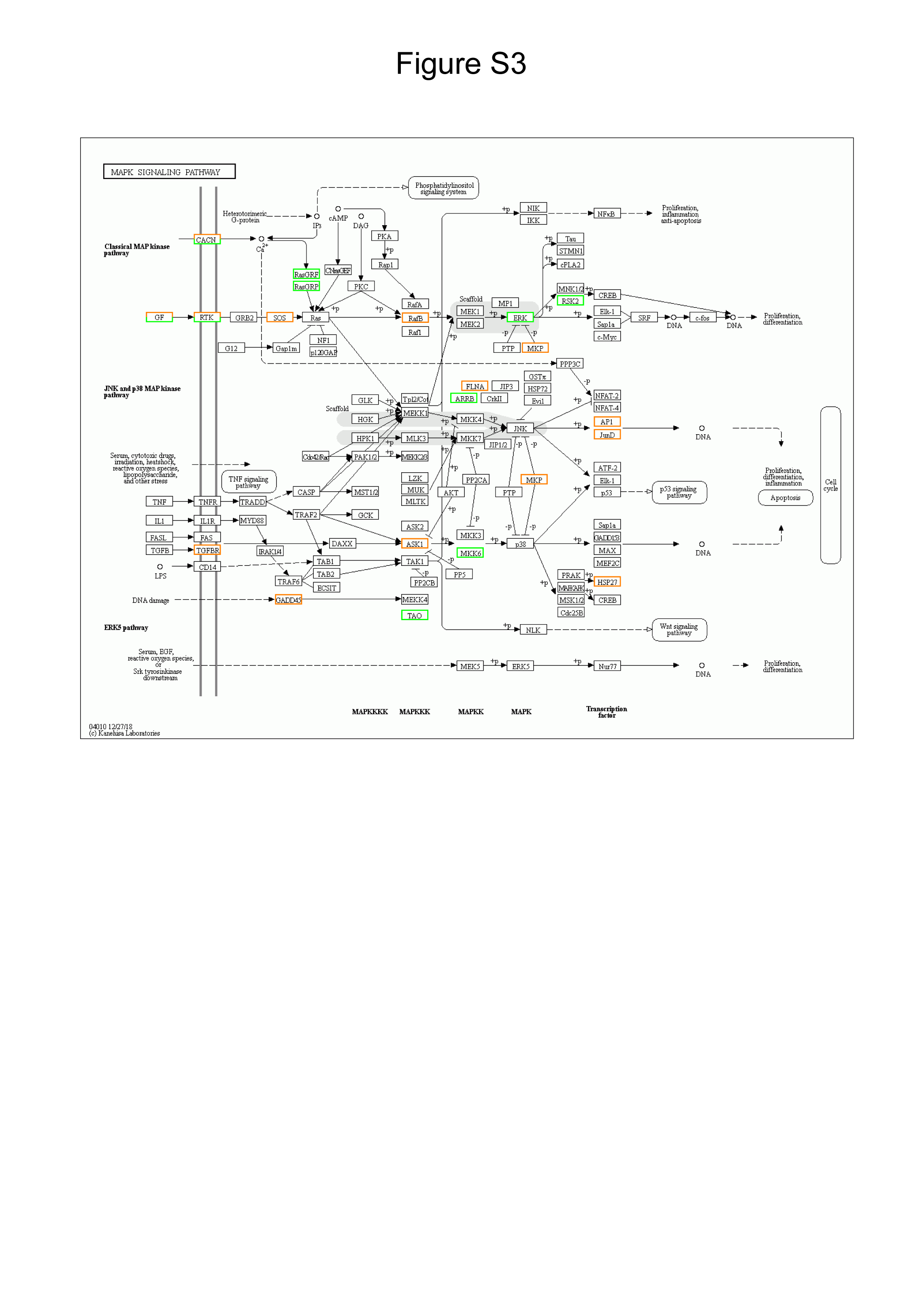

Supplement: Supplementary file 3 — The MAPK signal pathway diagram [file 41420_2021_667_MOESM3_ESM.tif]

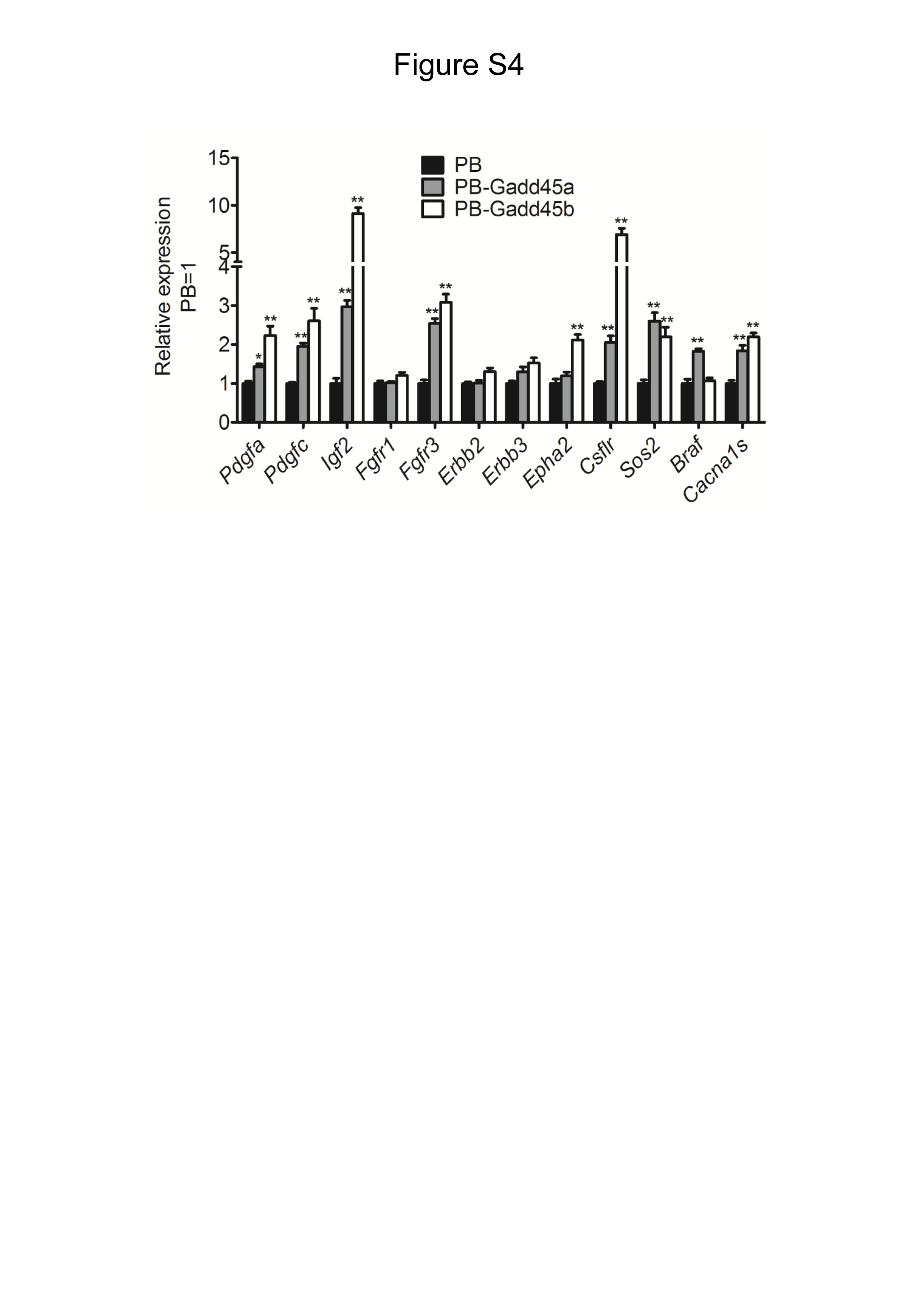

Supplement: Supplementary file 4 — Gadd45a and Gadd45b induce the expression of MAPK signaling pathway associated genes [file 41420_2021_667_MOESM4_ESM.tif]

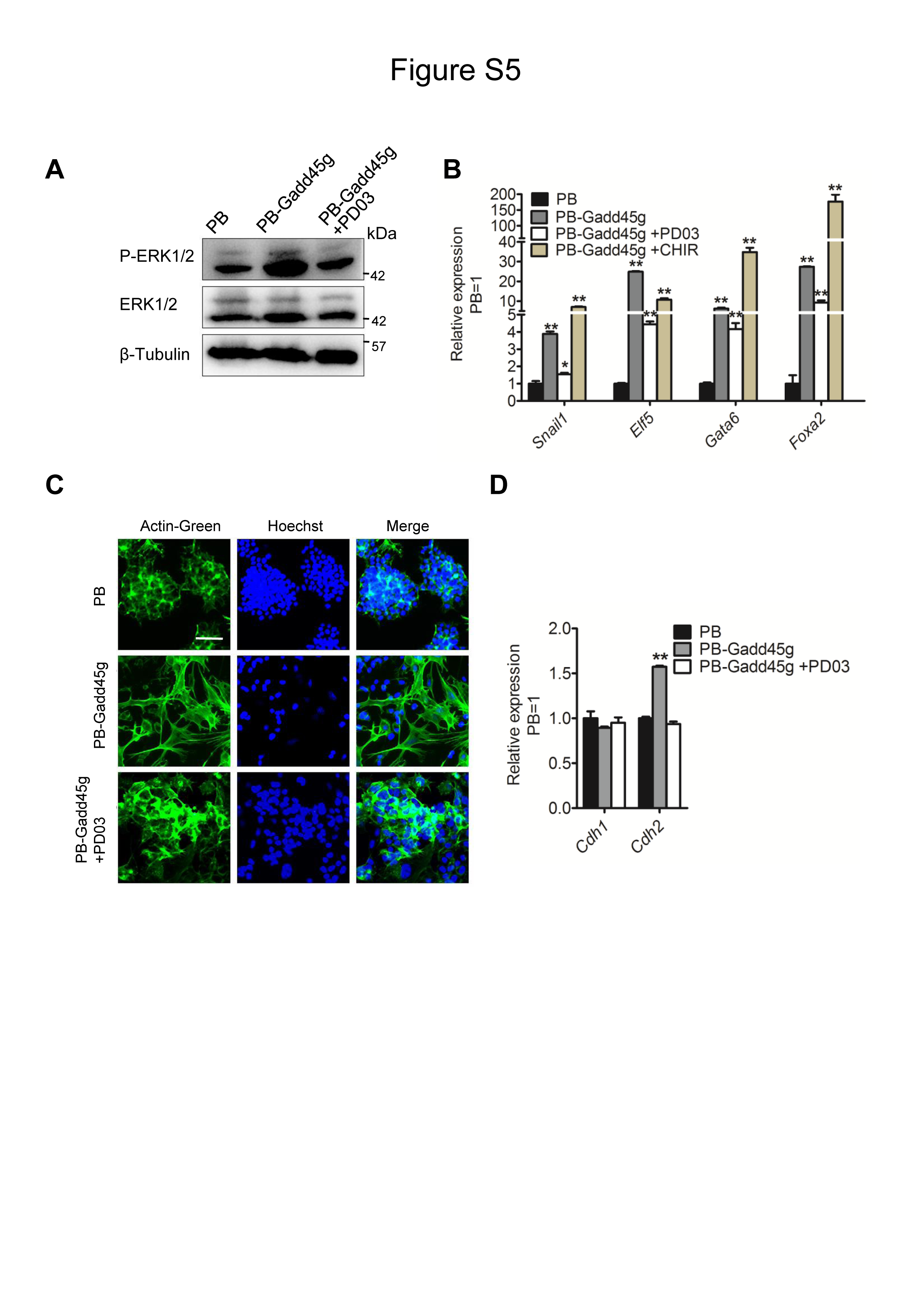

Supplement: Supplementary file 5 — Effects of PD03 on 46C mESCs overexpressing Gadd45g [file 41420_2021_667_MOESM5_ESM.tif]

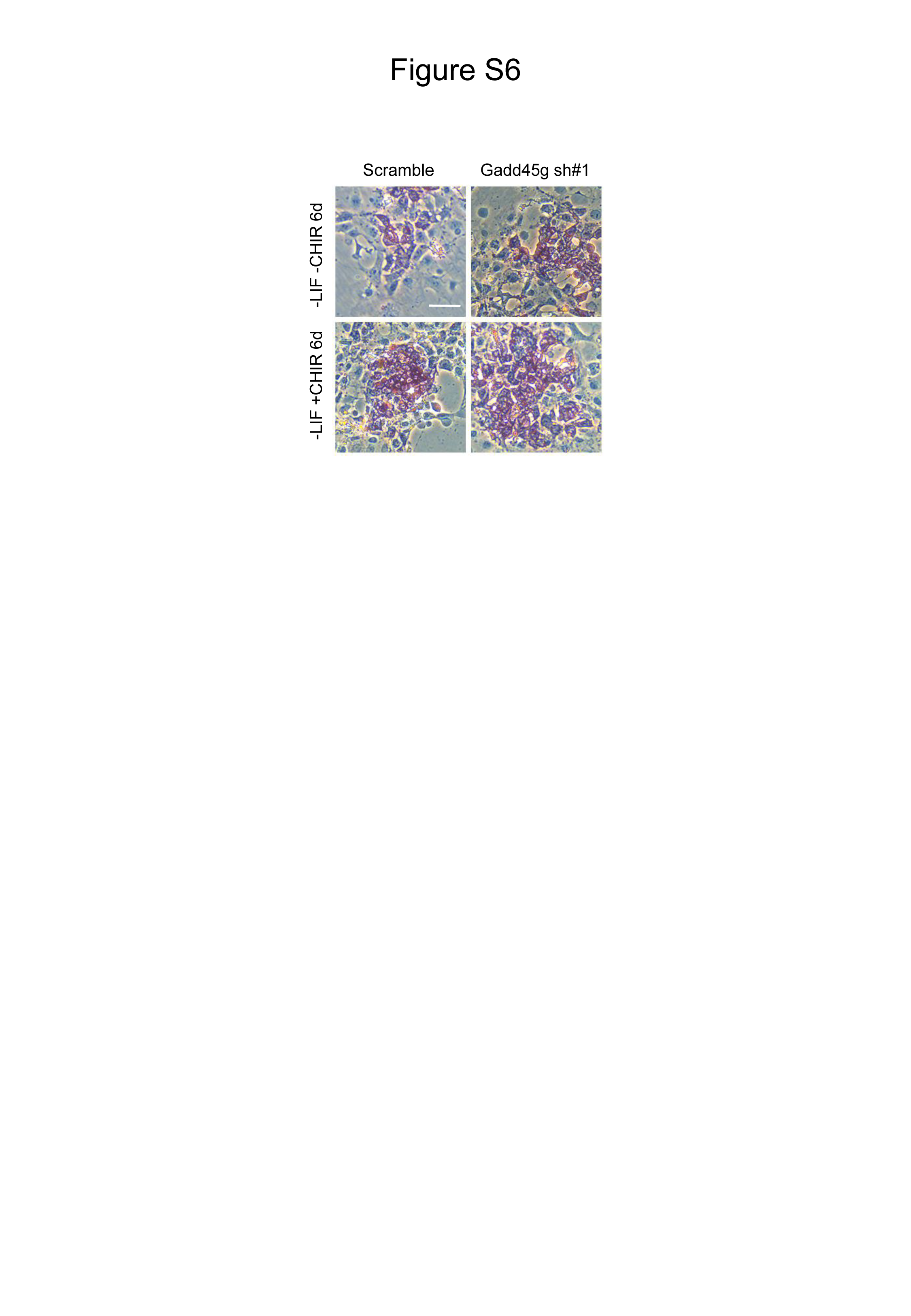

Supplement: Supplementary file 6 — CHIR fails to maintain stemness in Gadd45g shRNA mESCs [file 41420_2021_667_MOESM6_ESM.tif]

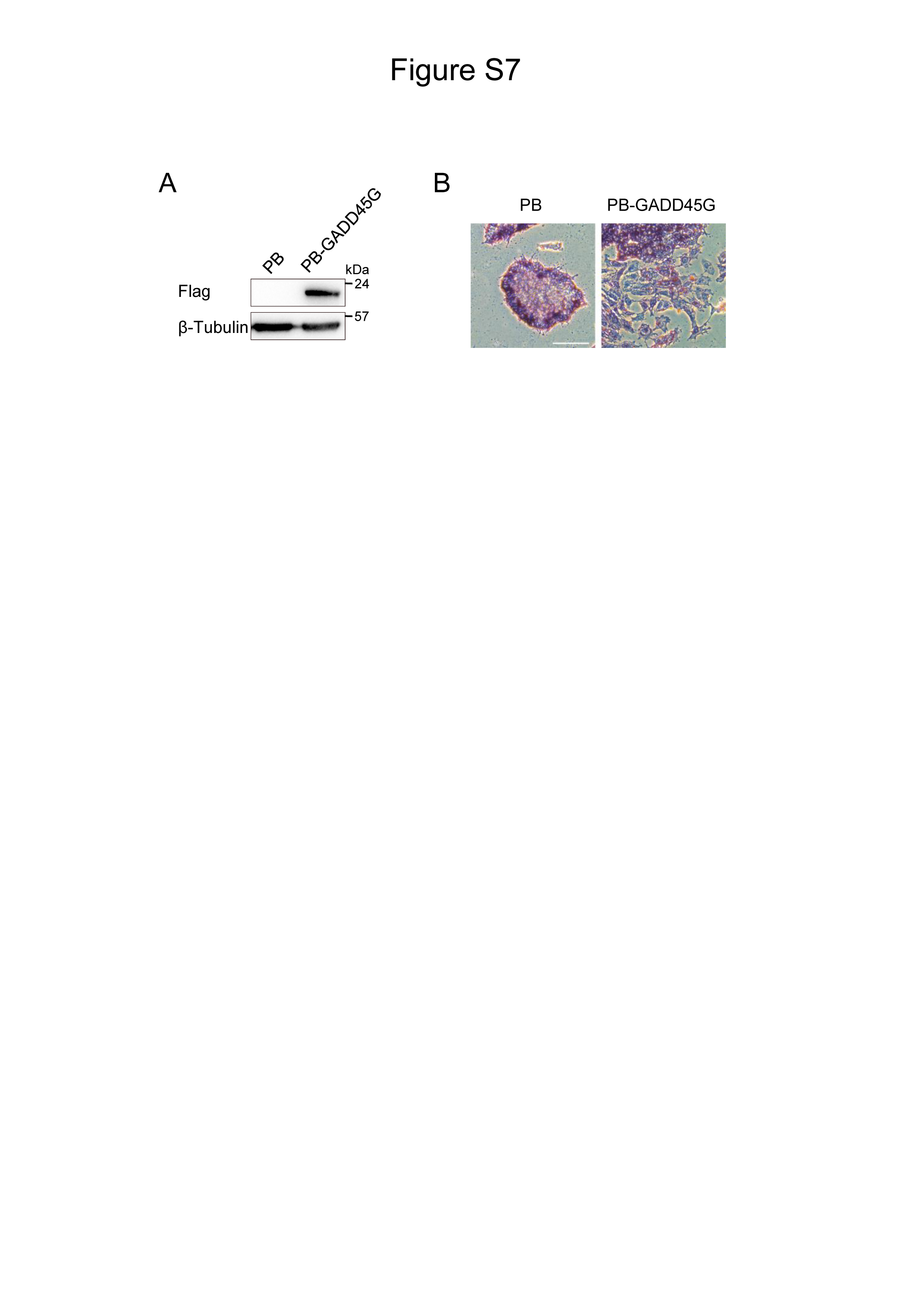

Supplement: Supplementary file 7 — . GADD45G overexpression promotes human iPSC differentiation [file 41420_2021_667_MOESM7_ESM.tif]

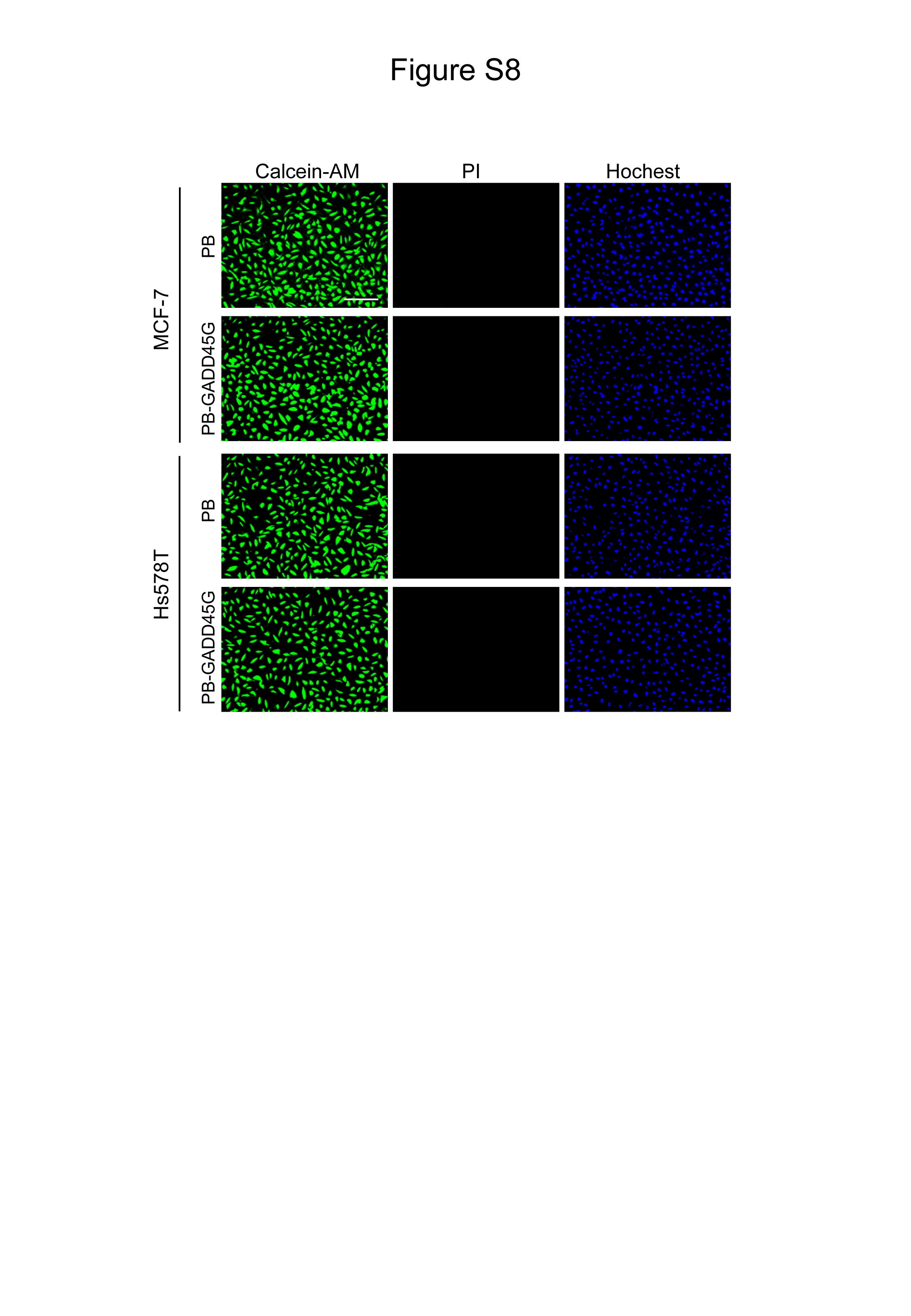

Supplement: Supplementary file 8 — The apoptosis of PB and PB-GADD45G breast cancer cells [file 41420_2021_667_MOESM8_ESM.tif]
