# Supplementary material for: Gadd45g initiates embryonic stem cell differentiation and inhibits breast cell carcinogenesis
Source: Cell Death Discov. 2021 Oct 2;7:271. doi: 10.1038/s41420-021-00667-x (PMC8487429; doi:10.1038/s41420-021-00667-x)
Supplement: Supplementary file 9 — List of primers for amplifying Gadd45 members [file 41420_2021_667_MOESM9_ESM.docx]

Table S1. List of primers for amplifying Gadd45 members

| Symbol | Forward sequence（5'-3'） | Reverse sequence（5'-3'） |
| --- | --- | --- |
| Mus musculus (house mouse) | | |
| Gadd45a | ATGACTTTGGAGGAATTCTCGG | TCACCGTTCCGGGAGATTAAT |
| Gadd45b | ATGACCCTGGAAGAGCTGGT | TCAGCGTTCCTCTAGAGAGATATAG |
| Gadd45g | ATGACTCTGGAAGAAGTCCGTGG | TCACTCGGGAAGGGTGATGC |
| Homo sapiens (human) | | |
| GADD45G | ATGACTCTGGAAGAAGTCCGCG | TCACTCGGGGAGGGTGATGC |
