# Supplementary material for: Gadd45g initiates embryonic stem cell differentiation and inhibits breast cell carcinogenesis
Source: Cell Death Discov. 2021 Oct 2;7:271. doi: 10.1038/s41420-021-00667-x (PMC8487429; doi:10.1038/s41420-021-00667-x)
Supplement: Supplementary file 10 — List of sequences used for gene knockdown [file 41420_2021_667_MOESM10_ESM.docx]

Table S2. List of sequences used for gene knockdown

| Symbol | ShRNA sequence（5'-3'） |
| --- | --- |
| Mouse Gadd45a sh#1 | GGATCCTGCCTTAAGTCAACT |
| Mouse Gadd45a sh#2 | GGAAAGTCGCTACATGGATCA |
| Mouse Gadd45b sh#1 | TGAAGAGAGCAGAGGCAATAA |
| Mouse Gadd45b sh#2 | GGCGGCCAAACTGATGAATGT |
| Mouse Gadd45g sh#1 | ATCCTCATTTCGAATCCTAAT |
| Mouse Gadd45g sh#2 | CATCCTCATTTCGAATCCTAA |
