# Supplementary material for: Gadd45g initiates embryonic stem cell differentiation and inhibits breast cell carcinogenesis
Source: Cell Death Discov. 2021 Oct 2;7:271. doi: 10.1038/s41420-021-00667-x (PMC8487429; doi:10.1038/s41420-021-00667-x)
Supplement: Supplementary file 11 — List of primers used for qRT-PCR analysis [file 41420_2021_667_MOESM11_ESM.docx]

Table S3. List of primers used for qRT-PCR analysis

| **Symbol** | | **Forward sequence（5'-3'）** | **Reverse order（5'-3'）** |
| --- | --- | --- | --- |
| Mus musculus (house mouse) | | | |
| RPL19 | GACGGAAGGGCAGGCATATG | | TGTGGATGTGCTCCATGAGG |
| Oct4 | AGAGGGAACCTCCTCTGAGC | | TTCATGTCCTGGGACTCCTC |
| Nanog | GTCTGATTCAGGGCTCAGCA | | AAGGCTTCCAGATGCGTTCA |
| Esrrb | TACCTGAACCTGCCGATTTC | | ATCTGGTCCCCAAGTGTCAG |
| Tfcp2l1 | AGGTGCTGACCTCCTGAAGA | | CAGGCTGTTATCCCCACTGT |
| Klf4 | CCAAAGAGGGGAAGAAGGTC | | CTGTGTGAGTTCGCAGGTGT |
| Gadd45a | TGAGCTGCTGCTACTGGAGA | | TGTGATGAATGTGGGTTCGT |
| Gadd45b | ACCCTGATCCAGTCGTTCTG | | TGACAGTTCGTGACCAGGAG |
| Gadd45g | GTTGATTCAGGCGTTCTGCT | | GCTCTCCTCGCAGAACAAAC |
| Gata4 | TCTCACTATGGGCACAGCAG | | GGGACAGCTTCAGAGCAGAC |
| Gata6 | TCCTCCCCTGCCGAAGTC | | AGGGCCAGAGCACACCAA |
| Foxa2 | CCTCAAGGGAGCAGTCTCAC | | TTTCTCCTGGTCCGGTACAC |
| Sox17 | TAAAGGTGAAAGGCGAGGTG | | CTTAGCTCTGCGTTGTGCAG |
| Nestin | CTCGAGCAGGAAGTGGTAGG | | TTGGGACCAGGGACTGTTAG |
| Otx2 | TCTGACCCCTTGTCCACTTC | | GAAGTTGAGCCAGCATAGCC |
| Fgf5 | GAAAAGACAGGCCGAGAGTG | | TGAACCTGGGTAGGAAGTGG |
| T | CCCTGCACATTACACACCAC | | CCCCTTCATACATCGGAGAA |
| Mixl1 | TTGAATTGAACCCTGTTGTCCC | | GAAACCCGTTCTCCCATCCACC |
| Cdx2 | AAGACAAATACCGGGTGGTG | | CCAGCTCACTTTTCCTCCTG |
| Elf5 | GGACCGATCTGTTCAGCAAT | | GCTGCCTCAATGAACTCCTC |
| Zeb1 | CCACTGTGGAGGACCAGAAT | | GATAGGGCTTTTCCCCAGAG |
| Zeb2 | CCGAGAAAGGACTGACAAGC | | CCCACAGCAATGGAGATTTT |
| Snail1 | TGAGAAGCCATTCTCCTGCT | | CTTCACATCCGAGTGGGTTT |
| Snail2 | TCTGCAGACCCACTCTGATG | | AGCAGCCAGACTCCTCATGT |
| Twist1 | TGAGCAAGATTCAGACCCTCA | | CATCTTGGAGTCCAGCTCGT |
| Mmp9 | TACAGGGCCCCTTCCTTACT | | TGCCTGTGTACACCCACATT |

Table S3. List of primers used for qRT-PCR analysis (Continued)

| **Symbol** | **Forward sequence（5'-3'）** | **Reverse order（5'-3'）** |
| --- | --- | --- |
| Cdh1 | AGTTTACCCAGCCGGTCTTT | TGTTGTGCTCAAGCCTTCAC |
| Cdh2 | GGGACAGGAACACTGCAAAT | CGGTTGATGGTCCAGTTTCT |
| Pdgfa | CAAGACCAGGACGGTCATTT | CCTCACCTGGACCTCTTTCA |
| Pdgfc | GTGCCAGGAAAGCAGACTTC | CACAGCATTGTTGAGCAGGT |
| Igf2 | TCCGAGAGGGACGTGTCTAC | CGTTTGGCCTCTCTGAACTC |
| Fgfr1 | CACCAAGAAGAGCGACTTCC | AACCAGGAGAACCCCAGAGT |
| Fgfr3 | GGATTTAGACCGCATCCTCA | GGGTGAACACCGAGTCATCT |
| Erbb2 | CCCATCAGAGTGATGTGTGG | GGGATCCCATCGTAAGGTTT |
| Erbb3 | CGCCAGATGACAAGCAGTTA | AGGTCATCAACTCCCAAACG |
| Epha2 | GCATTGTCATGTGGGAAGTG | ATCAAAGTCAGCCAGCGTCT |
| Csf1r | TTGGACTGGCTAGGGACATC | GGTTCAGACCAAGCGAGAAG |
| Sos2 | CAGTCCTCTTGCCACACTCA | GTGGAATAGCAGGAGGGTCA |
| Braf | GAATGTGACAGCACCCACAC | AAGTAATCCATGCCCTGTGC |
| Cacna1s | CGACCCAAAAAGGACACAGT TTCCTCCTCTGCTGTCAGGT | |
