# Supplementary material for: Gadd45g initiates embryonic stem cell differentiation and inhibits breast cell carcinogenesis
Source: Cell Death Discov. 2021 Oct 2;7:271. doi: 10.1038/s41420-021-00667-x (PMC8487429; doi:10.1038/s41420-021-00667-x)
Supplement: Supplementary file 12 — Supplementary figure legends [file 41420_2021_667_MOESM12_ESM.docx]

**Supplementary Figure legends**

**Figure S1. The expression levels of Gadd45 genes in EB cells**

Western blot analysis of Oct4, Gadd45a, Gadd45b and Gadd45g levels in 46C mESCs or EBs collected from different days. β-Tubulin was used as a loading control.

**Figure S2. Knockdown of Gadd45 family genes delay mESC differentiation**

(A-C) qRT-PCR analysis of Gadd45a, Gadd45b or Gadd45g expression levels in 46C mESCs infected with scramble, Gadd45a, Gadd45b or Gadd45g shRNA lentiviruses (Gadd45 sh#1 and Gadd45 sh#2). The data are represented as the means±s.d. (N=3 biological replicates). **p<0.01 vs scramble.

(D) qRT-PCR analysis of Gadd45a, Gadd45b and Gadd45g expression levels in 46C mESCs infected with scramble or Gadd45a/b/g shRNA lentiviruses (Gadd45 sh#1). The data are represented as the means±s.d. (N=3 biological replicates). **p<0.01 vs scramble.

(E) AP staining of scramble, Gadd45a, Gadd45b or/and Gadd45g shRNA mESCs cultured in serum-containing medium without LIF for 5 days and 10 days. d, days. Bar, 100 μM.

(F) qRT-PCR analysis of the expression of self-renewal genes (Tfcp2l1, Nanog and Oct4) in scramble, Gadd45a, Gadd45b, Gadd45g and Gadd45a/b/g shRNA mESCs cultured in serum-containing medium without LIF for 5 days. The data are represented as the means±s.d. (N=3 biological replicates). *p<0.05, **p<0.01 vs scramble.

**Figure S3. The MAPK signal pathway diagram**

Diagram of all genes involved in the MAPK signaling. Red, Gadd45g-upregulated genes ; Green, Gadd45g-suppressed genes.

**Figure S4. Gadd45a and Gadd45b induce the expression of MAPK signaling pathway associated genes**

qRT-PCR analysis of the indicated gene expression in PB, PB-Gadd45a or PB-Gadd45b 46C mESCs cultured in LIF/serum conditions. The data are represented as the means ± s.d. (N=3 biological replicates). *p<0.05, **p<0.01 vs PB.

**Figure S5. Effects of PD03 on 46C mESCs overexpressing Gadd45g**

(A) Western blot analysis of ERK1/2 and P-ERK1/2 levels in 46C mESCs overexpressing Gadd45g and treated with or without PD03. β-tubulin was used as a loading control.

(B) qRT-PCR analysis of Snail1, Elf5, Gata6 and Foxa2 gene expression levels in 46C mESCs overexpressing Gadd45g and treated with or without PD03 or CHIR. The data are represented as the means ± s.d. (N=3 biological replicates). *p<0.05, **p<0.01 vs PB.

(C) Actin-Tracker Green-488 fluorescent probe staining of 46C mESCs overexpressing Gadd45g and treated with or without PD03.

(D) qRT-PCR analysis of Cdh1 and Cdh2 gene expression levels in 46C mESCs overexpressing PB or PB-Gadd45g in the presence or absence of PD03. The data are represented as the means ± s.d. (N=3 biological replicates). **p<0.01 vs PB.

**Figure S6. CHIR fails to maintain stemness in Gadd45g shRNA mESCs**

AP staining of 46C mESCs infected with scramble or Gadd45g shRNA lentiviruses and cultured in serum-containing medium with or without CHIR for 6 days. d, days. Bar, 100 μM.

**Figure S7. GADD45G overexpression promotes human iPSC differentiation**

(A) Western blot analysis of FLAG in human iPSCs overexpressing FLAG-tagged human GADD45G (PB-GADD45G) or empty vector. β-tubulin was used as a loading control.

(B) AP staining of human iPSCs overexpressing PB or PB-GADD45G and cultured in mTeSR like medium ncTarget for 10 days. Bar, 100 μΜ.

**Figure S8. The apoptosis of PB and PB-GADD45G breast cancer cells**

The Calcein-AM and Propidium Iodide (PI) reagents were used to examine the apoptosis of breast cancer cells overexpressing PB or PB-GADD45G. Bar, 100 μΜ. Calcein-AM was used to stain live cells while PI was used to stain dead cells.
